# Prediction model to identify infectious COVID-19 patients in the emergency department

**DOI:** 10.1017/ash.2024.82

**Published:** 2024-05-17

**Authors:** Myat Oo Aung, Indumathi Venkatachalam, Jean X.Y. Sim, Liang En Wee, May K. Aung, Yong Yang, Edwin P. Conceicao, Shalvi Arora, Marcus A.B. Lee, Chang H. Sia, Kenneth B.K. Tan, Moi Lin Ling

**Affiliations:** 1 Infection Prevention and Epidemiology Department, Singapore General Hospital, Singapore, Singapore; 2 Department of Infectious Disease, Singapore General Hospital, Singapore, Singapore; 3 Emergency Department, Singapore General Hospital, Singapore, Singapore

## Abstract

**Background:**

Real-time reverse-transcriptase polymerase chain reaction (RT-PCR) has been the gold standard for diagnosing coronavirus disease 2019 (COVID-19) but has a lag time for the results. An effective prediction algorithm for infectious COVID-19, utilized at the emergency department (ED), may reduce the risk of healthcare-associated COVID-19.

**Objective:**

To develop a prototypic prediction model for infectious COVID-19 at the time of presentation to the ED.

**Material and methods:**

Retrospective cohort study of all adult patients admitted to Singapore General Hospital (SGH) through ED between March 15, 2020, and December 31, 2022, with admission of COVID-19 RT-PCR results. Two prediction models were developed and evaluated using area under the curve (AUC) of receiver operating characteristics (ROC) to identify infectious COVID-19 patients (cycle threshold (Ct) of <25).

**Results:**

Total of 78,687 patients were admitted to SGH through ED during study period. 6,132 of them tested severe acute respiratory coronavirus 2 positive on RT-PCR. Nearly 70% (4,226 of 6,132) of the patients had infectious COVID-19 (Ct<25). Model that included demographics, clinical history, symptom and laboratory variables had AUROC of 0.85 with sensitivity and specificity of 80.0% & 72.1% respectively. When antigen rapid test results at ED were available and added to the model for a subset of the study population, AUROC reached 0.97 with sensitivity and specificity of 95.0% and 92.8% respectively. Both models maintained respective sensitivity and specificity results when applied to validation data.

**Conclusion:**

Clinical predictive models based on available information at ED can be utilized for identification of infectious COVID-19 patients and may enhance infection prevention efforts.

## Introduction

In March 2020, coronavirus disease 2019 (COVID-19) was declared a pandemic by World Health Organization (WHO). On May 5, 2023, WHO announced that COVID-19 was no longer a public health emergency of international concern, with a global tally of 766 million cases and 7 million deaths on May 10, 2023.^1^

The pandemic gradually transitioned to endemicity with easing of community control measures and travel restrictions. However, within healthcare settings, efforts to prevent and control COVID-19 were necessitated for a longer period due to the vulnerability of the patient population and virulence of strains in circulation. Undifferentiated respiratory viral symptomatology and pre-symptomatic transmission capability of COVID-19 pose a challenge to infection prevention.

The gold standard for detecting severe acute respiratory coronavirus 2 (SARS-CoV-2), the causative agent for COVID-19, is by real-time reverse-transcriptase polymerase chain reaction (RT-PCR).^2^ RT-PCR test kits are comparable with high sensitivity, ranging from 99.4% to 99.5%.^3^ Cycle threshold (Ct) values provide guidance on infectiousness with Ct >24 being associated with low infectivity.^4^ However, RT-PCR is expensive and has a turnaround time of 2–3 hours, limiting its utility as a screening test in the emergency department (ED).

Point-of-care antigen rapid tests (ARTs) have been developed and utilized in ED settings in Singapore to triage and assign admission location. ARTs are known to have high false-negative rate, especially in patients who are asymptomatic with low viral load. Meta-analysis of 133 analytical and clinical studies showed pooled ART sensitivity of 71.2% (95% CI, 68.2%–74.0%).^5^ Bed allocation based on false-negative ART (ART−, PCR+) tests may result in inpatient exposures and secondary cases.

In Singapore, following transition to mitigation phase, active surveillance using SARS-CoV-2 RT-PCR and or ARTs was no longer routinely performed in ED settings. The role of prediction models that incorporate epidemiological risks and clinical parameters in the identification of infectious COVID-19 patients became important in prevention of healthcare-associated COVID-19.

Several COVID-19 prediction models that include standard laboratory tests, patient demographic data, exposure history, and symptoms have been developed and tested.^6,7^ A study conducted by Ducray *et al*. on the diagnostic performance of chest computed tomography showed sensitivity of 90.2% in detecting COVID-19.^8^ As previous studies on COVID-19 predictive models did not differentiate infectious COVID-19, we aimed to develop and assess prediction models for infectious COVID-19 (Ct <25) in our ED setting.

## Methods

### Setting

Singapore General Hospital (SGH) is an academic medical center in Singapore with 2,000 inpatient beds and specialized services including solid and hematopoietic stem cell transplant, oncology, and cardiothoracic surgery.

### Study design

Structured ED triage data and laboratory results from ED were extracted from hospital electronic system. Data were equally divided into training and validating data using stratified randomization. Ethics review exemption was granted by Institutional Review Board as this study used only anonymized data. No sampling of data was carried out as study included all patients admitted to SGH via ED with valid SARS-CoV-2 RT-PCR result (detected/not detected) during study period. As the aim of the study was to predict infectious COVID-19 cases with information available in ED setting, variables with >10% of missing data, laboratory tests which are not routinely performed at ED, were excluded from the analysis. We conducted retrospective cohort study including all adult patients admitted to SGH via ED, from March 15, 2020, to December 31, 2022, with valid ED SARS-CoV-2 RT-PCR results.

### COVID-19 risk management

Patients who were ART positive or high pretest probability for COVID-19 (positive epidemiological risk) were admitted to COVID-19 dedicated isolation wards pending PCR results and further clinical evaluation. Patients with negative ART and low pretest probability (no epidemiological risk) for COVID-19 were either admitted to general wards if they did not have acute respiratory infection (ARI) symptoms or respiratory surveillance wards (RSWs) if they had ARI symptoms.

On March 15, 2020, SGH ED started testing all patients with epidemiological risk (contact of COVID-19-positive person, visit to known COVID-19 cluster areas, overseas travel, under government risk restriction orders (quarantine order, stay-home notice, etc)) for COVID-19 and ARI symptoms (cough, runny nose, sore throat, loss of taste/smell, shortness of breath), using RT-PCR. From June 2021 to October 2022, all patients admitted through ED had to undergo screening using both an ART and a SARS-CoV-2 PCR test. ART results, available within 15–30 min, were incorporated into a decision algorithm that included epidemiological risks and clinical presentation, to risk stratify patients and determine their admission location.

During the study period, when COVID-19-infected inpatients were identified in non-isolation multi-bedded wards, they were immediately isolated in COVID-19-designated isolation wards, and environmental disinfection was conducted. Contact tracing was done to identify at-risk contacts who were then placed on enhanced surveillance (RT-PCR test on days 1 and 4 and daily ART for 5 days from the last date of exposure).

### COVID-19 diagnostic tests

**Figure 1.**
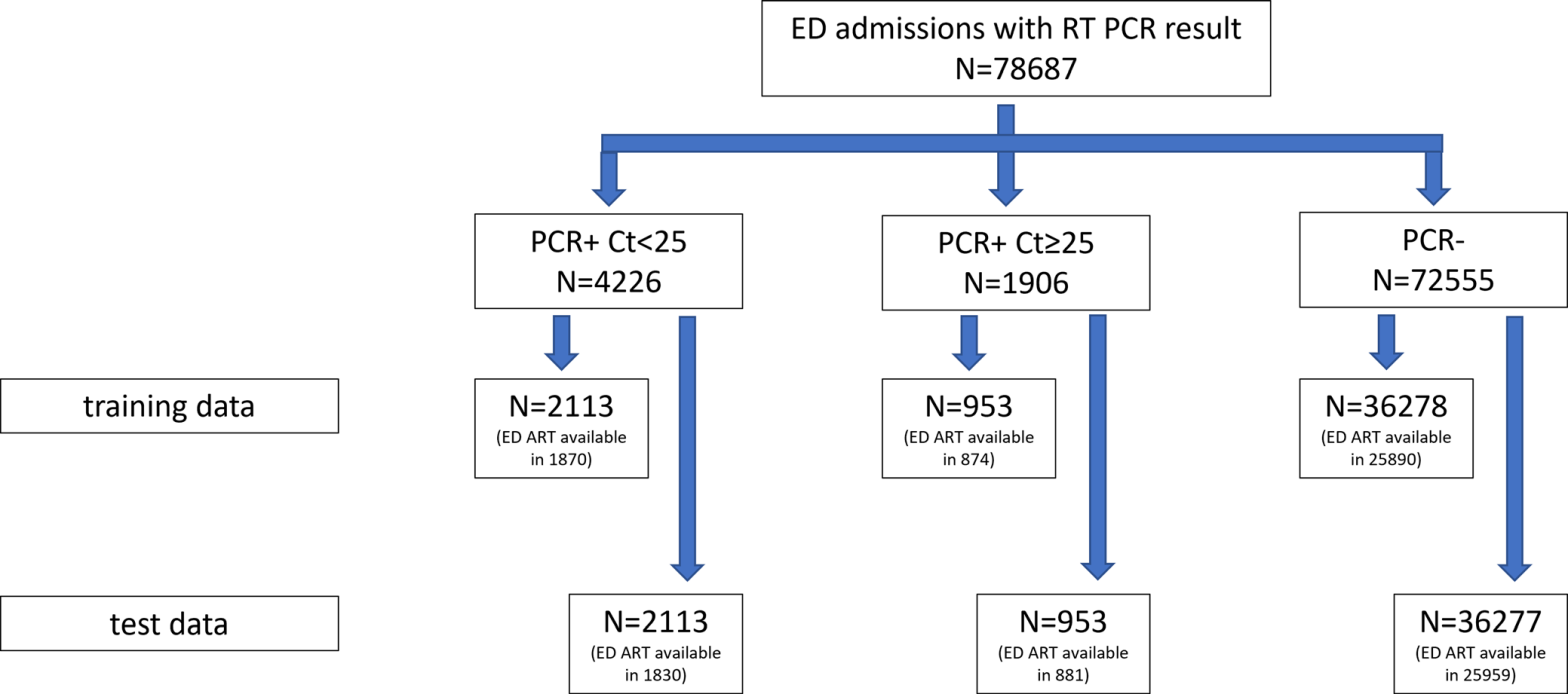
Study population.

RT-PCR was done using the Cepheid GeneXpert Xpress SARS-CoV-2 test, intended for the quantitative detection of nucleic acid from SARS-CoV-2, targeting the N2 and E gene regions of SARS-CoV-2. Ct values for both N2 and E genes are reported. Patients with SARS-CoV-2 PCR Ct value <25 for either N2 or E genes are classified as having infectious COVID-19. Patients with SARS-CoV-2 PCR Ct value ≥25 for both N2 and E genes are classified as having none infectious COVID-19. Patients with SARS-CoV-2 PCR not detected are grouped together with none infectious COVID-19 for analysis (Figure 1).

ART kits used in SGH during study period were as follows: BD Veritor System for Rapid Detection of SARS-CoV-2 (May 24, 2021–June 30, 2022), SD Biosensor Standard Q COVID-19 Antigen Rapid Test (December 2, 2021–June 30, 2022), Abbott Panbio COVID-19 Antigen Rapid Test (October 13, 2021–June 30, 2022), and Flowflex SARS-CoV-2 Antigen Rapid Test (March 11, 2022–June 30, 2022). Brands of ART kits used by patients for self-testing were not documented in hospital system.

### Variables included in the analyses

Patient demographic, laboratory results, medical history, symptoms, and clinical condition at ED presentation were included in the prediction algorithm. These variables were selected based on data availability and previous reports on COVID-19 risk predictors.^6–9^ Patients who had received government COVID-19 restriction orders were electronically tagged in the hospital electronic medical records (EMRs) from which all required data were extracted.

### Statistical analyses

Statistical analyses were performed using IBM Statistical Package for Social Science (SPSS) V.26. Categorical variables were analyzed using χ^2^ test, although parametric continuous variables were compared with *t* test. Categorical variables were coded into 1 (yes) and 0 (no). Variables that showed *P* values of <0.10 by univariate analysis were included in the model building. Multiple logistic regression with backward stepwise elimination was used to identify clinical predictors of infectious COVID-19. Variables included in the model were tested for multicollinearity using Pearson correlation coefficient. No strong correlation was observed among these variables. Model performances were evaluated using area under the curve (AUC) of receiver operating characteristics (ROCs). Two models were tested. Model 1 had demographic, medical history, symptom variables, and laboratory test results. Model 2 included all variables from model 1 as well as ART result at ED for subset of the study population. Optimal cutoff value of predicted probability for these models was selected using Youden’s index with consideration toward higher sensitivity.

Mathematical equations were built to calculate predictive probability for both models using variables’ beta values and applied to test period data. Individual patient’s predictive probability of infectious COVID-19 was calculated and classified using optimal cutoff value. Sensitivity and specificity of both models were further calculated using RT-PCR result as gold standard.

## Results

From March 15, 2020, to December 31, 2022, total of 78,687 patients were admitted to SGH through ED. Of these admissions, 6,132 (6.4%) patients tested positive for COVID-19 with SARS-CoV-2 RT-PCR. The highest monthly number of SARS-CoV-2 PCR+ patients (1,019 cases, 23%) was observed during the pandemic wave in March 2022. Of 6,132 PCR+ patients, nearly 70% (4226) of these patients had infectious COVID-19 (Ct <25) (Figure 1).

Comparing the characteristics of infectious COVID-19 patients with combined noninfectious COVID-19- or PCR-negative patient groups, older age, Chinese race, high C-reactive protein (CRP), low platelets, low white blood cell (WBC) count, and hyponatremia were significantly associated with infectious COVID-19. Patients with history of exposure to COVID-19 (direct contact or visit to COVID-19 cluster areas), self-test ART positive within 72 hours of ED visit, and those under any government COVID-19-related restriction orders had significant risk association with infectious COVID-19. Infectious COVID-19 cohort had significantly higher proportion of symptomatic patients with fever, cough, sore throat, and runny nose. They were also more likely to have tachycardia, shortness of breath, tachypnea, and required supplemental oxygen (Table 1). CRP, procalcitonin, lactate dehydrogenase, and ferritin had to be excluded from the model analysis due to high missing value (>10%) as these tests were not routinely done in ED. ED’s ART results were available for 57,304 patients (72.8% of the study population) as ART testing was performed for all patients in ED only from May 2021 onward. Patients tested ART positive at ED were significantly associated with infectious COVID-19 (Table 1).

**Table 1.** Characteristic of the study population

	PCR+ Ct <25 (N = 4226)	PCR– or Ct ≥25 (N = 74461)	OR (95% CI)	*P* value*
**Demographic**				
Gender (male)	2195 (51.9)	39141 (52.6)	0.9 (0.9–1.0)	0.428
Age (year)	68.9 (17.5)	64.8 (17.8)	1.01 (1.01–1.02)	<0.001
Chinese	3170 (75.0)	52529 (70.5)	1.2 (1.1–1.3)	<0.001
**Laboratory**				
High CRP	2903 (73.1)	28402 (67.4)	1.3 (1.2–1.4)	<0.001
Low platelet count	614 (14.6)	6269 (8.5)	1.8 (1.6–2.0)	<0.001
Low WBC count	449 (10.7)	3035 (4.1)	2.8 (2.5–3.1)	<0.001
Hyponatremia	1392 (33.1)	18178 (24.7)	1.5 (1.4–1.6)	<0.001
**History**				
Contact with COVID-19-positive person	67 (1.6)	198 (0.3)	6.0 (4.5–7.9)	<0.001
Self-tested ART positive within preceding 72 hours	2128 (50.4)	287 (0.4)	262 (230–298)	<0.001
Traveled abroad within preceding 14 days	9 (0.2)	96 (0.1)	1.6 (0.8–3.2)	0.145
Under government risk restriction order**	159 (3.8)	293 (0.4)	9.8 (8.1–12.0)	<0.001
Transferred from nursing home, care home, other hospitals	162 (3.8)	2638 (3.5)	1.1 (0.9–1.2)	0.321
**Symptoms and clinical conditions**				
Fever	1754 (41.5)	12604 (16.9)	3.5 (3.2–3.7)	<0.001
Cough	2007 (47.5)	10635 (14.3)	5.4 (5.1–5.8)	<0.001
Sore throat	985 (23.3)	1637 (2.2)	13.5 (12.4–14.7)	<0.001
Runny nose	775 (18.3)	1663 (2.2)	9.8 (8.9–10.8)	<0.001
Loss of taste and smell	22 (0.5)	53 (0.1)	7.3 (4.5–12.1)	<0.001
Shortness of breath	489 (11.6)	5103 (6.9)	1.8 (1.6–1.9)	<0.001
Tachycardia	908 (22.5)	13693 (18.8)	1.3 (1.2–1.4)	<0.001
Required supplementary oxygen	308 (7.3)	3401 (4.6)	1.6 (1.4–1.8)	<0.001
Tachypnea	416 (10.3)	6204 (8.5)	1.2 (1.1–1.4)	<0.001
ART tested positive at ED	3346 (90.4)	343 (0.6)	1467 (1260–1709)	<0.001

Note. PCR, polymerase chain reaction; Ct, cycle threshold; CRP, C-reactive protein; WBC, white blood cell; COVID-2019, coronavirus disease 2019; ART, antigen rapid test; ED, emergency department.

Categorical data are shown in number (%). Continuous data are shown in mean (SD).

* Pearson χ^2^ test (categorical data); *t* test (continuous data).

** Government risk restriction orders (quarantine order, stay-home notice, health risk warning, health risk alert).

High CRP, CRP >9.1 mg/L; low platelet count, platelet <140×10^9^/L; low WBC count, WBC<4.0×10^9^/L; hyponatremia, NA <135 mmol/L; tachycardia, heart rate of >100 beats/min; tachypnea, respiratory rate >20/min

**Figure 2.**
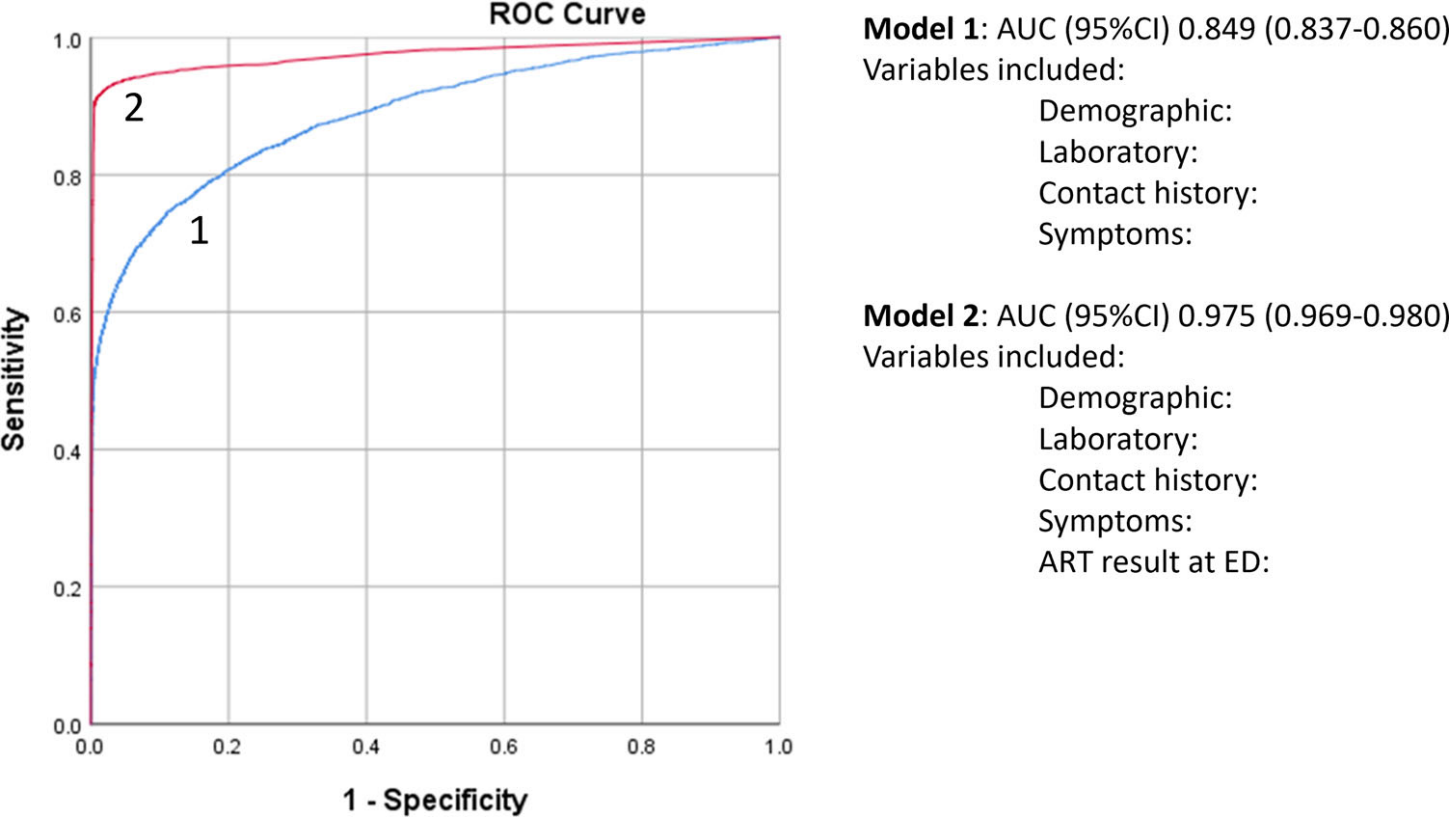
Area under the curve of different prediction models.

Data were split equally into training data set and validation data set using stratified randomization. Training data had a total of 39,344 patients, and validation data had a total of 39,343 patients (Figure 1). Model 1, which included demographic, laboratory results, history, and symptoms (Table 2), yielded AUROC (95% CI) of 0.85 (0.84–0.86). When cutoff threshold was defined at 0.00005994, using Youden’s index, model 1 had sensitivity and specificity of 80.0% and 72.1%, respectively. Model 2, which incorporated ART results at ED (Table 2), yielded AUROC (95% CI) of 0.97 (0.96–0.98). When cutoff threshold was defined at 0.0005793, model 2 had sensitivity and specificity of 95.0% and 92.8%, respectively. Paired-Sample Area Difference test shows model 2 has significantly higher AUROC over model 1 (*P*<0.001) (Figure 2).

**Table 2.** Variables included in prediction models

	Model 1*			Model 2*		
	Beta	OR (95% CI)	*P* value	Beta	OR (95% CI)	*P* value
**Demographic**						
Chinese				0.220	1.24 (0.96–1.61)	0.097
Age (year)	−0.019	0.98 (0.97–0.98)	<0.001			
**Laboratory**						
Low platelet count (platelet<140×10^9^/L)				0.311	1.36 (0.95–1.96)	0.091
Low WBC count (WBC<4.0x10^9^/L)	0.753	2.12 (1.70–2.64)	<0.001			
Hyponatremia (NA<135mmol/L)	0.350	1.42 (1.24–1.61)	<0.001	0.282	1.33 (1.03–1.70)	0.027
**History**						
Contact with COVID-19-positive person	1.251	3.49 (2.03–5.99)	<0.001	1.341	3.82 (1.11–13.20)	0.034
Self-tested ART positive within preceding 72 hours	4.861	129 (106–157)	<0.001	0.974	2.65 (1.85–3.79)	<0.001
Under government risk restriction order	1.531	4.62 (2.86–7.45)	<0.001	1.759	5.81 (2.27–14.85)	<0.001
**Symptoms**						
Fever	0.771	2.16 (1.89–2.46)	<0.001	0.669	1.95 (1.50–2.53)	<0.001
Cough	0.855	2.35 (2.06–2.68)	<0.001	0.748	2.11 (1.62–2.75)	<0.001
Sore throat	1.440	4.22 (3.46–5.14)	<0.001	0.806	2.24 (1.47–3.39)	<0.001
Runny nose	1.107	3.02 (2.45–3.73)	<0.001			
Loss of taste and smell	1.263	3.53 (1.16–10.75)	0.026			
Shortness of breath	0.527	1.69 (1.39–2.06)	<0.001	0.351	1.42 (0.95–2.11)	0.083
Required supplementary oxygen	0.215	1.24 (0.98–1.56)	0.073	0.522	1.68 (1.05–2.69)	0.029
Tachycardia (heart rate>100/min)						
Tachypnea (respiratory rate>20/min)						
**ART tested positive at ED**				6.696	809 (631–1037)	<0.001
Intercept	−9.243			−8.867		

Note. WBC, white blood cell; COVID-2019, coronavirus disease 2019; ART, antigen rapid test; ED, emergency department.

* Multiple logistic regression with backward stepwise elimination. Variables without beta value are eliminated by model.

**Figure 3.**
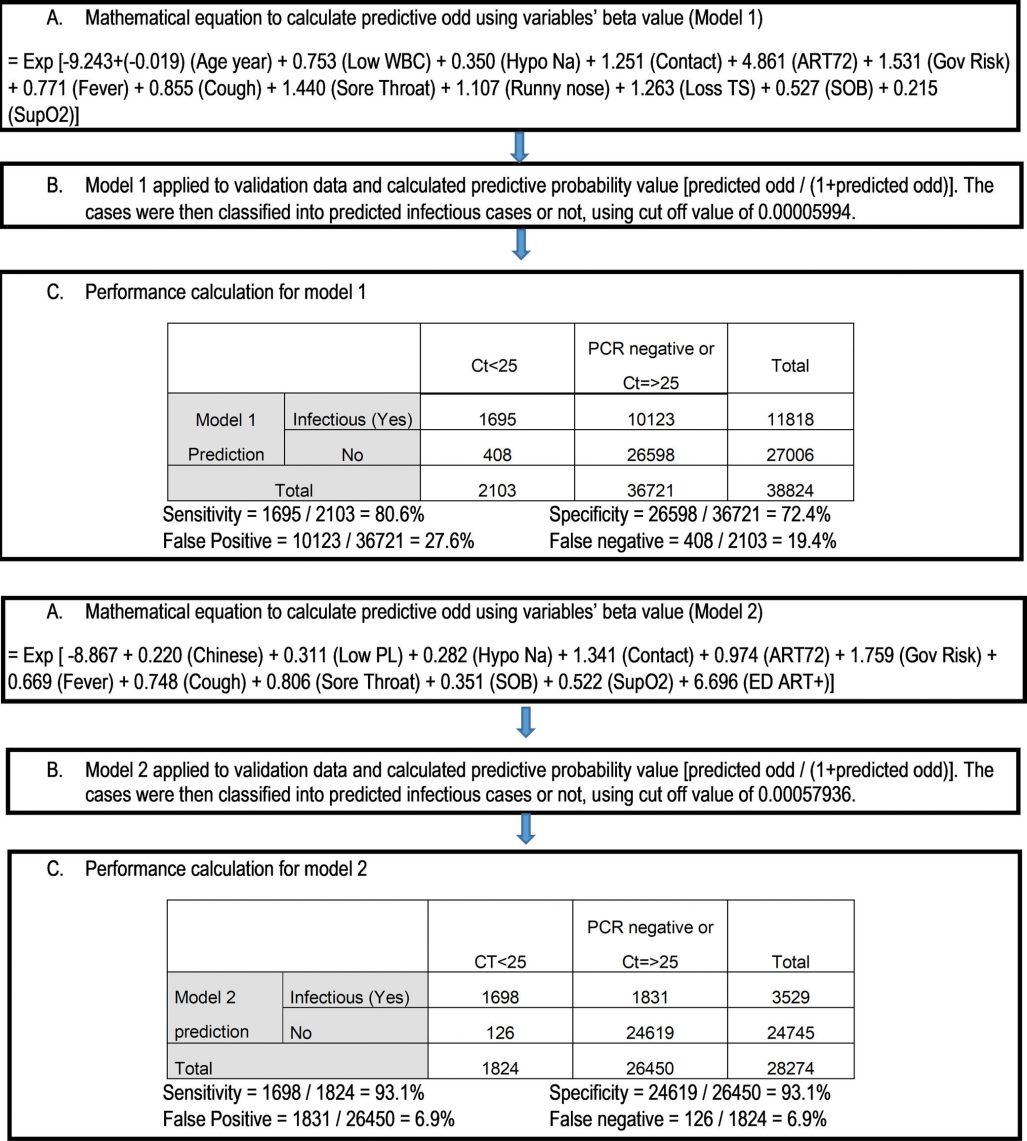
Calculation process of predictive probability and performance for model 1 and model 2.

Individual patient’s predictive probability of infectious COVID-19 was calculated using mathematical equation of both models and applied to the validation data. For model 1, predicted probability value of 0.00005994 was used to classify infectious COVID-19. Patients who had predictive probability equal to or above this value were classified as having infectious COVID-19. Similarly, for model 2, cutoff value of 0.0005793 was used. Using RT-PCR result as gold standard, model 1 achieved sensitivity and specificity of 80.6% and 72.4%, respectively, on the test data. Model 2, which included ED ART result, achieved sensitivity and specificity of 93.1% and 93.1%, respectively, on the test data (Figure 3).

## Discussion

Clinical predictors of COVID-19 have been identified in several studies. A prediction model that includes only standard laboratory tests yielded sensitivity and specificity of 82.4% and 86.8%, respectively.^6^ A model that includes patient demographic data, laboratory, and symptoms achieved high performance with AUROC of 0.97 to predict COVID-19-positive cases in a German study.^7^ In the COVID-19 endemic state, screening for COVID-19 using RT-PCR and ART upon hospital admission may not be cost-effective. Our model without ART in ED performed less favorably with AUROC of 0.89 (sensitivity and specificity of 70.6% and 91.7%, respectively) versus 0.95 (sensitivity and specificity of 91.3% and 99.1%, respectively) with addition of ART.

The lower performance of our model without ART results may be due to exclusion of some variables with high missing data and longer study period where weightage of individual variable may have changed over time. Furthermore, our model aims to detect infectious COVID-19 cases instead of all positive COVID-19. Although elevated CRP has been observed in COVID-19 patients,^9^ CRP was excluded from the model analysis due to high missing value (>10%). Similarly, for procalcitonin, lactate dehydrogenase, and ferritin, data were not available at ED level for most of the study population. Thrombocytopenia and leucopenia were observed in COVID-19 patients.^10,11^ Low platelet counts and low WBC counts were also associated with infectious COVID-19 in our study and included in the predictive model.

Other studies have also reported risk predictions for COVID-19. Male, African American race, older age, and those with known COVID-19 exposure were at higher risk of COVID-19. But reduced risk was observed among influenza-vaccinated persons.^12^ Mamidi *et al*. used credit scorecard modeling approach to estimate the probability of COVID-19. International Classification of Disease information available in the electronic health record was used in predicting risk of COVID-19, and the model achieved high AUC of 0.84.^13^ Chew *et al*. developed risk prediction scores to identify patients with low risk of COVID-19 based on demographic, clinical symptoms, exposure risks, and blood test results. The study based on patients admitted to RSW in single center for duration of 3-month period; two models were developed using logistic regression method and achieved AUROC of 0.934 and 0.866, respectively. Authors claimed that during study period, if these two models had been employed, 20%–40% of patients with low risk of COVID-19 would not have been subjected to respiratory surveillance. Isolation days and PCR tests for these patients could have been avoided.^14^

COVID-19 vaccines have been effective at preventing severe disease, hospitalization, and death but not infection. Vaccines may have had reduced effectiveness for emerging variants such as what was observed with the Omicron variants.^15–17^ As of January 2023, 83% of Singapore population achieved minimum protection status with completion of 3 doses of mRNA Novavax/Nuvaxovid vaccine or 4 doses of Sinovac-CoronaVac vaccines.^18^ As most of Singapore’s population had been vaccinated, vaccination status may have had minimum impact on predicting COVID-19 and hence was not incorporated in our algorithm.

Limitations of our study include missing data, longer study period, and changing characteristics of emerging COVID-19 variants that may have affected the efficiency of our predictive models. Being retrospective study, based on available data collected at ED triage record, some important clinical, comorbidity information and family history are missing in our study. Prospective structured data collection of clinical variables that have been proven to be associated with COVID-19, dynamic predictive model that regularly adapts to changing clinical presentations of new COVID-19 variants, could have increased model efficiency. Although both models were validated using test data at SGH, external validation is required to assess applicability to other settings.

Unlike previous studies, our study focused on detecting infectious COVID-19 with limited information available at ED setting. We have identified predictive models that include demographic, laboratory results, history, symptom variables, and point-of-care test result, which can be utilized to identify infectious COVID-19 during triage at ED setting. The regression formula can easily be implemented for prediction probability calculation at ED setting using commonly available software such as Microsoft Excel.

## Conclusion

Our study has shown that the clinical information available at ED setting can be utilized in a statistical model and enhance prediction of infectious COVID-19. As most of the data elements are already available through hospital’s EMRs, a validated predictive model can be an important tool in pandemic preparedness planning within healthcare facilities.

Together with clinical judgment, these predictive models can be an important tool for determination of admission location and help reduce misplacement of infectious COVID-19 cases to non-isolation ward facilities, in particular prior to the availability of point-of-care test kits for emerging infections.
